# Eating out intensity, ultra-processed foods and BMI among Albanian youth

**DOI:** 10.1017/S1368980023002173

**Published:** 2023-12

**Authors:** Ferenc Vincze, Taulant Muka, Fabian Eichelmann, Erand Llanaj

**Affiliations:** 1 Department of Public Health and Epidemiology, Faculty of Medicine, University of Debrecen, Debrecen, Hungary; 2 Institute of Social and Preventive Medicine, University of Bern, Bern, Switzerland; 3 Meta-Research Innovation Centre at Stanford (METRICS), Stanford University, Stanford, CA, USA; 4 Epistudia, Bern, Switzerland; 5 Department of Molecular Epidemiology, German Institute of Human Nutrition Potsdam-Rehbrücke, Arthur-Scheunert-Allee 114-116, Nuthetal, 14558, Germany; 6 German Centre for Diabetes Research (DZD), München-Neuherberg, Germany

**Keywords:** Ultra-processed foods, Eating out of home, Youth, BMI, Diet quality

## Abstract

**Objective:**

Ultra-processed foods (UPF) and eating out of home (OH) are changing nutrition, particularly among youth in constrained settings. We aimed to assess the role of eating OH intensity on the associations of UPF and unprocessed or minimally processed foods (UMPF) with BMI among Albanian youth.

**Design:**

Cross-sectional.

**Setting:**

Albania, a south-eastern European country.

**Participants:**

281 youth, predominantly females.

**Methods:**

UPF and UMPF were defined based on NOVA, while eating OH intensity based on energy percentage from OH foods. Multivariable models tested associations of UPF and UMPF with BMI stratified by eating OH intensity, controlled for relevant covariates including diet quality, portion size and costs.

**Results:**

The respondents age ranged between 18 and 23 years with a female predominance (87·5 %). Mean energy from UPF and UMPF was 846 (sd: 573·0) and 802·9 (422·5) kcals, respectively. Among substantial at home eaters UPF intake was not associated (*β* = −0·07, 95 % CI (−0·13, 0·267)) with BMI; however, UMPF negatively associated with BMI (*β* = −0·24, 95 % CI (−0·43, −0·06)). Among those defined as substantial OH eaters, UPF (*β* = 0·24, 95 % CI (0·08, 0·40)) and UMPF (*β* = 0·18, 95 % CI (0·04, 0·33)) were positively associated with BMI.

**Conclusions:**

Our findings provide evidence for the hypothesis that eating OH plays an important role in the association of UPF and UMPF with BMI in youth. While causality cannot be established due to cross-sectional design, to the best of our knowledge, we provide the first assessment of UPF and UMPF intake in a south-eastern European setting, while highlighting the need for establishing and integrating youth nutrition into national nutritional surveillance systems for key dietary risk factors in Albania.

## Graphical abstract

**Figure:**
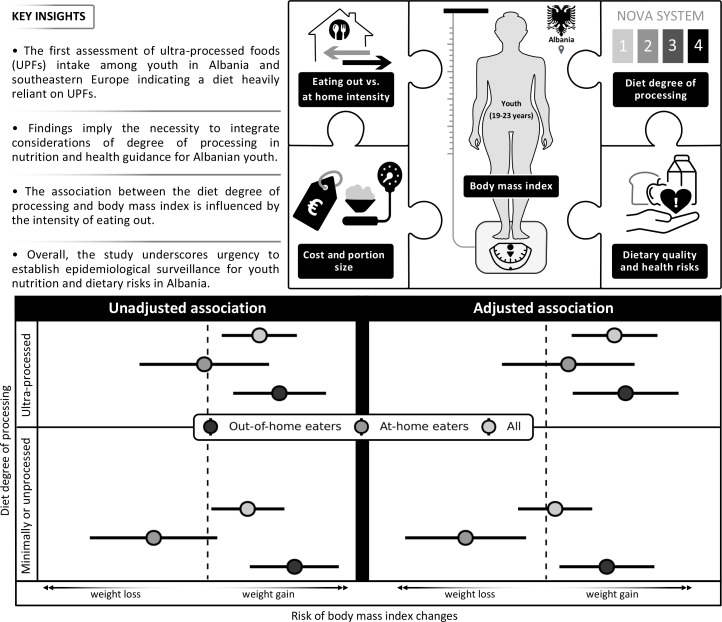

Consumption of ultra-processed foods (UPF) has been strongly associated with poor diet quality^(1)^, greater risk of diet-related noncommunicable diseases^(2,3)^, cancer^(4)^ and all-cause mortality^(5)^. UPF consumption accounts for over a half of total energy intake in multiple countries and is rapidly increasing worldwide^(6)^, particularly among younger age groups^(7,8)^. There is evidence that these trends are associated with detrimental shifts in body size and composition^(9,10)^ and *ad libitum* intake^(11)^ in this age group. In contrast, consumption of unprocessed or minimally processed foods (UMPF) has been associated with favourable patterns of protein intake, diet quality and lower cardiometabolic risks^(12)^.

Youth – the transition from adolescence to adulthood – is a time of rapid changes in physical growth and development, as well as cognitive and emotional capacities^(13)^. While there has been a great emphasis on early childhood nutrition in the field, it is important to recognise that youth phase also presents risks and opportunities, accompanied with long-term and intergenerational consequences.

Yet, youth nutrition has been overlooked in the UN Decade of Action on Nutrition (2016–2025) and the Sustainable Development Goals for nutrition, as they do not contain any youth-specific nutrition targets^(14)^. The Global Action Plan of the WHO for the prevention and control of noncommunicable diseases also lacks clearly specified targets for overweight and obesity in youth^(15)^. Despite the fact that overweight and obesity among youth more than doubled globally from 1990 to 2016 and the number of young girls with anaemia increased by 20 %^(16)^, investments in and research for youth nutrition remain woefully inadequate. As day follows night, diet-related noncommunicable diseases inevitably follow overweight and obesity, highlighting the importance of scrutinising the role of UPF consumption in youth nutrition and disease prevention. This urgency is reinforced by high-quality meta-analyses of large prospective cohort studies that have demonstrated a robust association between UPF consumption and the risk of type 2 diabetes^(17)^.

Accumulating evidence indicates that consumption of UPF and convenience meals have increased recently^(18)^, alongside a parallel trend and rapid expansion of eating out of home (OH). This has been partially fuelled by the digitalisation and commercialisation of youth health and well-being on social media^(19)^, particularly in the WHO European Region^(20)^. Consumption of UPF has been linked to eating location^(21)^, raising concerns about the link between eating OH intensity and UPF. This link becomes especially important when considering the impact of UPF not only on human but also on environmental health and sustainability^(22)^.

Recognising that accelerated global action is needed to address the pervasive and corrosive burden of malnutrition in all its forms, the Global Nutrition Report regularly reports on progress in nutrition for all countries and regions. The latest report shows that not a single country in the south-eastern Europe is on course to meet the targets for obesity among men and women^(23)^. In line with Global Nutrition Report, the latest Global Burden of Disease study on health effects of dietary risks estimated that a relatively high proportion of deaths among adults in this part of Europe in 2017 were attributable to dietary risks (i.e. 32 % (95 % CI (29·7, 34·3))^(24)^.

Among youth, data are scarce, virtually non-existent in Albania (i.e. an upper middle-income south-eastern European country of the Mediterranean basin). The latest ‘*2022 Report of the European Commission’* has highlighted the critical state of malnutrition in Albania and the pressing need to develop a nutrition plan and the urgency to raise awareness on dietary risks^(25)^. To the best of our knowledge, there has been no quantified dietary intake data available for youth or other population groups in Albania in the past 30 years, including data on UPF consumption. The only quantified dietary intake data available for Albania have been published recently by our research group^(26,27)^. In these studies, we have indicated an extremely poor adherence to established standards and guidelines for health and environmental sustainability, while highlighting the distressing presence of dietary risks.

Here, we conduct the first analysis on UPF and UMPF consumption in relation to eating OH intensity and evaluate their association with BMI, taking into consideration food costs and diet quality indices considered healthy and sustainable among youth in Albania.

## Methods

The present analysis is based on observational data collected cross-sectionally in Albania^(26)^.

### Study design and sample

Dietary intake data were collected using a single multiple pass 24-h dietary recall and processed with Lucille Food Intake platform hosted by Ghent University. The 24-h dietary recall was conducted using the validated multiple pass method described in our previous work^(26)^. In brief, the instrument uses a standardised methodology to make recall of all possible foods as accurate as possible and address recall bias. In addition, students of Master of Public Health, at the University of Medicine Tirana, were recruited and trained on the use of 24-h instrument. The study included young adults (18–23 years) sampled from the three largest universities in Albania (36–38 % of county’s undergraduate students were studying there during data collection), namely University of Tirana, University of Medicine Tirana and Polytechnic University of Tirana.

To be eligible for inclusion in the study, participants had to be enrolled in an undergraduate programme at one of the aforementioned universities. This criterion intended to exclude students enrolled in postgraduate programmes, who were often working whilst studying and were thus not in regular contact with the typical food environment. However, some special programmes were included despite of this restriction, since they had an integrated master of science programme (e.g. medicine, pharmacy, dentistry and architecture).

In brief, 364 participants were initially invited. A total of thirty-five participants reported that the recall day was not representative of a typical day and were therefore excluded. Of the remaining 326 participants, eleven were excluded due to health issues (i.e. flu, common cold, etc.) on the day prior or during the past 24 hours. Further, twenty-six participants wanted to do the recalls, but did not give their consent for their recall information to be included in our analyses and were therefore not considered further. A more detailed description of study design, validation of the recall instrument and analysis of the food environment have been published elsewhere^(26,27)^.

**Fig. 1:**
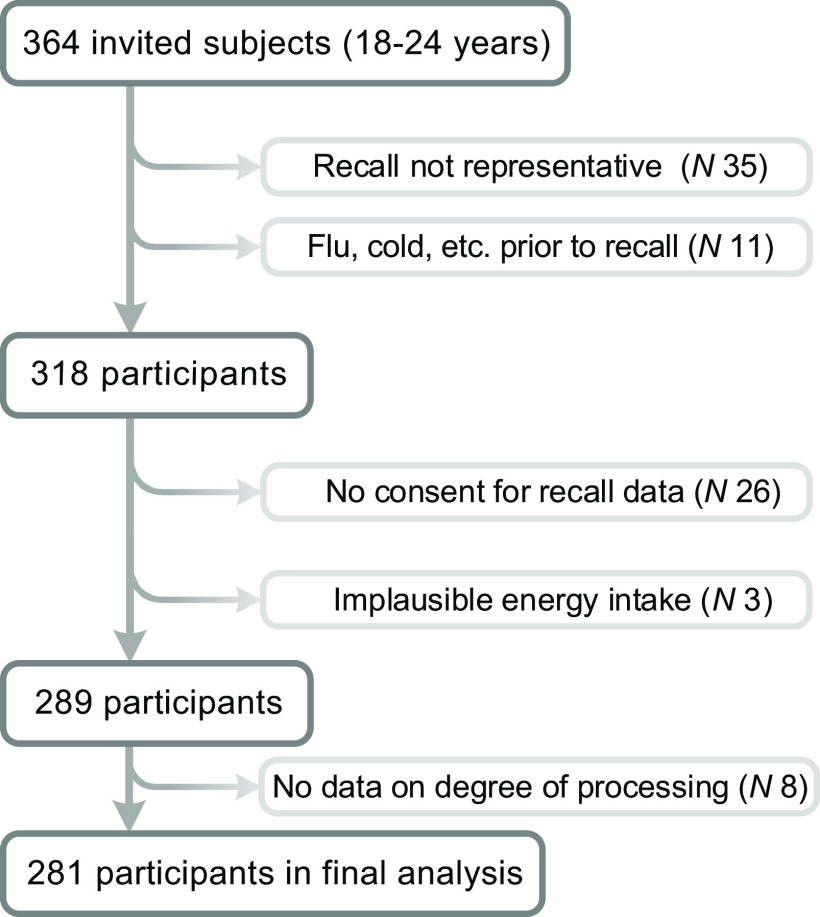
Flow diagram for the selection of participants included in the final analysis

A priori high and low sex-specific cut-off points were established for plausible energy intake and three cases that fell outside of the cut-offs were excluded. Eventually, 289 remaining participants were considered (response proportion: 79·4 %). During the data-cleaning process, the sample was further restricted to only those respondents with valid and non-missing data for UPF (excluding eight participants), resulting in a final sample of 281 participants for data analysis presented in this report (Fig. 1). Sensitivity analysis of the excluded participants showed that their exclusion did not affect the investigated associations (not shown).

### Ethical approval

All participants were informed before giving their written consent and participating in the study. The study protocol and methods were approved by the Medical Ethics Committee of Ghent University Hospital (No. EC/2015/1118), the Directorate of Health Care at the Ministry of Health and Social Protection in Albania (No. MSH/2015/LL-13-1) and the Ethics Committee of the University of Medicine Tirana (No. ELL-2016) in line with the Declaration of Helsinki guidelines.

### Degree of processing, composition and food cost data

All food and drink items were classified according to the NOVA system into UMPF and UPF^(28)^. UMPF were those that were altered only by removing inedible or unwanted parts, drying, crushing, grinding, fractionating, filtering, roasting, boiling, pasteurisation, refrigeration, freezing, placing in containers, vacuum packaging or non-alcoholic fermentation, without adding any other ingredients. A few examples include fresh fruits and vegetables, boiled eggs and untreated nuts or seeds. UPF were identified when food substances never or rarely used were found among the list of ingredients. Some of those substances include different types of sugars (fructose, high fructose corn syrup, ‘fruit juice concentrates’, inverted sugar, maltodextrin, dextrose and lactose), modified oils (hydrogenated or inter-esterified oils), protein sources (hydrolysed proteins, soy protein isolate, gluten, casein, whey protein and ‘mechanically separated meat’), as well as additive cosmetics used for aroma, flavour enhancers, dyes, emulsifiers, among other applications. Some examples include energy drinks, packaged snacks (e.g. chips and most crackers) and processed meats like cold cuts or sausages.

Data on food intake were coupled with price data of each individual food item to estimate diet cost. Prices in Albanian LEK were obtained from local fast-foods, supermarkets, restaurants and other food vendors around the university facilities where the data collection was performed and were converted in Euro (€) based on the conversion rate at the time the study was conducted (i.e., 1 € = 13,521 Albanian LEK).

### Definition of eating out of home

Eating OH may be defined by either the place of consumption or source of food. In the literature, ‘*eating out of home’* and ‘*away from home eating*’ tend to be used interchangeably. Both concepts refer to the same notion of practices, involving foods and drinks prepared OH. We considered OH foods to include foods that were not prepared at home and were obtained near fast-foods, restaurants, street food vendors and other OH sources of food, including food products purchased ready-to-eat from food stores, such as supermarkets, convenience stores and some special food market. To define eating OH intensity, participants were classified as ‘*substantial at home (AH) eaters*’ if equal to or less than 30 % of their total dietary energy intake came from foods and drinks prepared OH and as ‘*substantial OH eaters’* if this percentage was higher than 30 %.

### Dietary indices and scores

Adherence to the Mediterranean diet score was computed according to the KIDMED index developed by Serra-Majem and colleagues for subjects up to 24 years old^(29)^. The index is based on a sixteen question test and the score ranges between 0 and 12, founded on principles that sustain a Mediterranean dietary pattern, as well as those that undermine it. Questions denoting a negative connotation with respect to the Mediterranean diet were assigned a value of −1 (e.g. eating at a fast-food restaurant, takes sweets and candy several times every day) and those with a positive aspect +1 (e.g. use of olive oil, likes pulses and eats them more than once a week). The sums of the values can be interpreted as three levels of adherence: (1) ≥8, optimal Mediterranean diet; (2) 4–7, improvement needed to adjust intake to Mediterranean patterns and (3) ≤3, very low diet quality and adherence to Mediterranean dietary pattern.

The DASH score was calculated as previously done by Mellen and colleagues based on nutrient targets derived from DASH trials (i.e., targets for saturated fat, total fat, protein, cholesterol, fibre, Mg, Ca, potassium and Na)^(30)^. In our analyses, individuals who met the goal for each component received 1 point (e.g. protein up to 18 % of total energy or Mg no < 238 mg/1000 kcal), those who met an intermediate goal (e.g. saturated fats up to 11 % of total energy intake or cholesterol below 107·1 mg/1000 kcal), defined as the midpoint between the DASH diet goal and the nutrient content of the DASH control diet received 0·5 point and those who met neither goal received 0 points. A total score was generated by considering all nutrient components, resulting in a minimum of 0 and a maximum of nine points.

WHO guidelines for the prevention of chronic diseases^(31)^ and the WHO 2020 Updated Healthy Diet Fact Sheet^(32)^ were used to construct a modified version of the Healthy Diet Indicator, using seven nutrient standards (i.e., SFA, PUFA, total protein, total dietary fibre, monosaccharides and disaccharides, cholesterol and potassium). Further, individual scores were summed, and participants received a maximum Healthy Diet Indicator score of seven points, if all Healthy Diet Indicator targets were met and a minimum of 0 points if none was met (final scores ranged between 0 and 7).

Dietary intake data were also compared with the EAT-*Lancet* reference diet for healthy diets from sustainable food systems^(33)^. The EAT-*Lancet* score (0–14 points) was calculated based on adherence to fourteen key dietary recommendations, as described by Knuppel *et al*.^(34)^

### Anthropometric assessment

A mechanical scale was used for weight and a simple measuring tape for height. Height and weight were measured under the supervision and assistance of trained interviewers, and for reliability, the measurements were taken in duplicate. A third measurement was performed if the first two measurements differed by > 200 g for weight and > 2 cm for height. BMI was calculated as weight (in kilograms) divided by height (in meters) squared.

### Statistical analysis

Descriptive analysis was presented using the computed means and standard deviation (sd) of the relevant variables. We compared the participants’ characteristics by two eating OH intensity categories, i.e. *substantial AH v*. *substantial OH eaters* using Mann–Whitney test.

Spearman’s correlation was performed to measure the strength and direction of monotonic association between variables used in regression models. Multivariate linear regression analyses were performed in two steps for exploring the associations between energy intake from UPF and UMPF-based energy intake and BMI. Distribution of variables with a non-normal distribution was normalised by Box–Cox transformation. The minimally adjusted (model 1) model testing the association between BMI (outcome) and UPF and UMPF (exposures) was controlled for age and sex. For the same association, a fully adjusted multivariable model (Model 2) was additionally controlled for diet quality scores, portion sizes and diet costs. Associations were quantified by standardised regression coefficients (*β*) and the corresponding 95 % CI. Sensitivity analyses of sex as a potential moderator of the association between UPF/UMPF consumption and BMI among youth OH *v*. AH settings showed negligible effects (not shown).

Statistical analysis was performed using *R* software version 4.0.5. We report results in accordance with STROBE (*STrengthening the Reporting of OBservational studies in Epidemiology*) extension for nutrition and dietary assessment^(35)^.

## Results

### Participant characteristic

Table 1 summarises the respondents’ characteristics. There was a female predominance (i.e., males: 12·5 % *v*. females: 87·5 %), and more than 60 % of the sample was characterised as substantial OH eaters. The mean (±sd) BMI was 21·3 kg/m^2^ (±2·8), and mean energy intake from UMPF and UPF was 802·9 kcals (±422·5) and 846 kcals (±573), respectively. Mean energy intake from UPF was significantly higher among substantial OH eaters (mean_OH_ = 1020·9 kcals, sd: ±602·6) compared with substantial AH eaters (mean_AH_ = 543·6 kcals, sd: ±354·4). Adherence to Mediterranean diet (mean_AH_ = 5·8, sd: ±1·9; mean_OH_ = 3·6, sd: ±2·0) and DASH (mean_AH_ = 4·0, sd: ±1·2; mean_OH_ = 3·2, sd: ±1·1) were significantly higher among substantial AH eaters. Further statistically relevant differences could not be found between the two groups (Table 1).

**Table 1:** Dietary, anthropometric and socio-economic characteristics of substantial at-home and out of home eaters

Observed variable	Overall (*n* 281)			Substantial at home eaters (*n* 103)		Substantial out of home eaters (*n* 178)		*P* value^*^
	Mean	sd	Range	Mean	sd	Mean	sd	
Age (years)	19·7	1·2	18–23	19·7	1·3	19·7	1·1	0·812
BMI (kg/m^2^)	21·3	2·8	16–33·8	20·9	2·5	21·5	2·9	0·124
Energy intake from UPF (kcal)	846	573	0–3961·5	543·6	354·4	1020·9	602·6	<0·001
Energy intake from UMPF (kcal)	802·9	422·5	0–2281·5	814·6	415·4	796·1	427·6	0·755
Mediterranean diet score (0–12)	4·4	2	0–11	5·80	1·90	3·61	2·03	<0·001
EAT-*Lancet* score (0–14)	1·65	1·02	0–5	1·81	1·11	1·56	0·96	0·084
HDI score (0–7)	3·5	1·3	1–6	3·6	1·2	3·5	1·3	0·540
DASH score (0–9)	3·5	1·2	0·5–7	4·0	1·2	3·2	1·1	<0·001
Portion size (g)	1527·6	413·3	144–3087	1575·6	394·4	1499·8	422·4	0·119
Food cost (€)	5·97	2·1	2·1–16·9	5·7	1·8	6·1	2·2	0·202

DASH, dietary approaches to stop hypertension; HDI, healthy diet indicator; UPF, ultra-processed foods; UMPF, unprocessed or minimally processed foods.

* Mann–Whitney *U* test for differences between at-home *v*. out of home eaters.

**Fig. 2:**
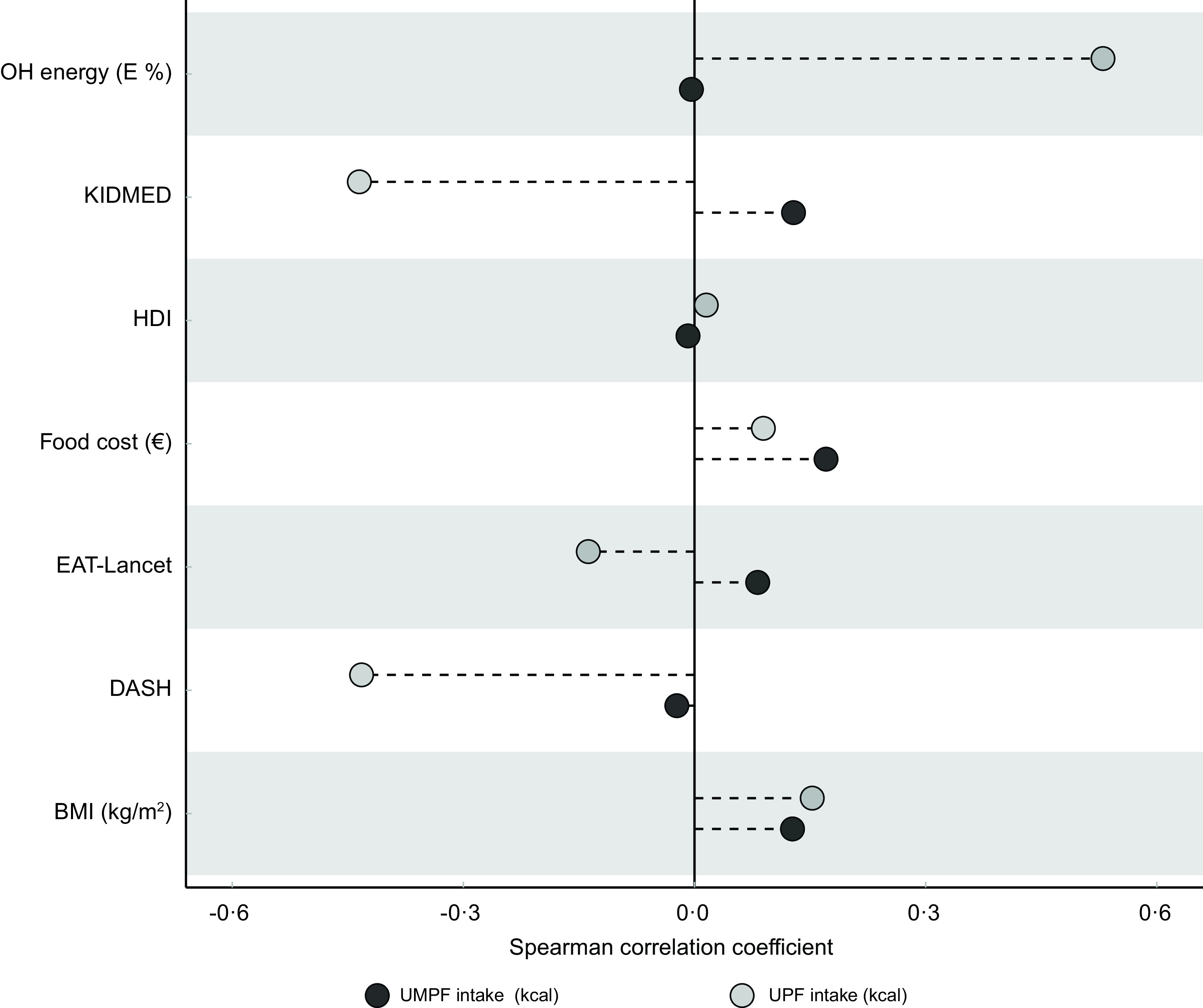
Correlation between main variables used in the analyses. HDI, healthy diet indicator score; KIDMED, Mediterranean diet score; DASH, Dietary Approaches to Stop Hypertension score; UPF, ultra-processed foods; UMPF, unprocessed or minimally processed foods; E %, contribution as percentage of energy from total calories

In Fig. 2, eating OH intensity was strongly and positively correlated with UPF intake. Mediterranean diet score and DASH diet score were negatively correlated with UPF intake. Correlations of the rest of the variables with UMPF or UPF intake were weak or negligible.

### Association of ultra-processed and unprocessed/minimally processed foods intake with BMI

Overall, a strong positive association between energy intake coming from UPF and BMI was observed both in minimally adjusted (*β* = 0·16, 95 % CI (0·04, 0·27)) and fully adjusted multivariable model (*β* = 0·21, 95 % CI (0·08, 0·34)) (Table 2). For UMPF, overall, there was a significant association with BMI in the minimally adjusted model (*β* = 0·12, 95 % CI (0·01, 0·23)), but it did not persist in the fully adjusted model for UMPF (*β* = −0·03, 95 % CI (−0·08, 0·14)).

**Table 2:** Ultra-processed foods and BMI among substantial at-home and out of home eaters

	Total sample				Substantial at-home eaters				Substantial out of home eaters				
	Model 1^†^		Model 2^‡^		Model 1		Model 2		Model 1		Model 2		
	*β*	95 % CI	*β*	95 % CI	*β*	95 % CI	*β*	95 % CI	*β*	95 % CI	*β*	95 % CI
UPF intake (kcal)	0·16	0·04, 0·27^*^	0·21	0·08, 0·34^*^	−0·01	−0·21, 0·19	0·07	−0·13, 0·27	0·22	0·08, 0·36^*^	0·24	0·08, 0·40^*^
UMPF intake (kcal)	0·12	0·01,0·23^*^	−0·03	−0·08, 0·14	−0·16	−0·36, 0·03	−0·24	−0·43, −0·06^*^	0·26	0·13, 0·40^*^	0·18	0·04, 0·33^*^

UPF, ultra-processed foods; UMPF, unprocessed or minimally processed foods.

* Significant predictor (*P* < 0·05).

*Note*:

† Model 1 is adjusted for sex, age and energy intake.

‡ Model 2 is additionally controlled for diet quality, portion size and food costs.

All values are given as: standardised linear regression coefficients (*β*) with the corresponding 95 % CI unless otherwise indicated.

Among substantial AH eaters, the only significant association observed was in the fully adjusted multivariable model, indicating an inverse association between UMPF intake (*β* = −0·24, 95 % CI (−0·43, −0·06)) and BMI. No additional significant associations were observed in this subgroup for UPF or UMPF and the BMI as an outcome.

Among substantial OH eaters, the minimally adjusted model showed that energy intake from both UPF (*β* = 0·22, 95 % CI (0·08, 0·36)) and UMPF (*β* = 0·26, 95 % CI (0·13, 0·40)) was significantly associated with increased BMI. These associations for both UPF (*β* = 0·24, 95 % CI (0·08, 0·40)) and UMPF (*β* = 0·18, 95 % CI (0·04, 0·33)) with BMI persisted in the fully adjusted model.

## Discussion

During the transition to adulthood, youth’s main influences on diet gradually shift from mainly parents and family to schools, peers and friends, as well as food marketing and broader social forces. The present study provides new insights for youth nutrition and a first assessment on UPF and UMPF intake in south-eastern Europe, specifically Albania. The intensity of eating OH modifies the association between UPF/UMPF consumption with BMI. Those who have higher percentage of energy coming from OH had almost double the energies from UPF. This may mean that those who eat more OH consume meals rich in UPF and a higher BMI might be more strongly associated with UPF consumption than those who eat less OH. Intensity of eating OH can modify the association between UPF intake and BMI, by potentially amplifying UPF intake, and consequently, its impact on BMI. While individual choices and the broader food environment unquestionably exert significant influence on dietary patterns, it in noteworthy that a higher intake of UMPF was inversely associated with BMI among AH eaters.

To the best of our knowledge, there are currently no published data on the consumption of UPF among youth in the Balkans and/or south-eastern Europe that can be compared with our results. On this note, south-eastern European nations share cultural and historical connections, yet each holds distinct socio-economic, dietary and lifestyle traits affecting UPF consumption. Our findings might not fully be transferable to all these nations, but they offer more relevant insights than findings extrapolated from Western/Central Europe or the United States, given the differing contexts. However, studies conducted on adults in Europe have shown a dramatic increase in the consumption of UPF in recent years, with UPF potentially contributing up to half of total daily energy intake in many countries^(36,37)^. For example, a survey conducted in Italy between 2010 and 2013 found that UPF made up of nearly a quarter of total daily energy intake among children and young adults^(38)^. In concert, these data suggest that UPF are displacing long established dietary patterns and have become a major part of diets in Europe.

Moreover, the results of this study are in line with previous research of higher methodological rigor that has found strong associations between UPF consumption and increased risk of obesity. For example, the only randomised controlled feeding study on diets rich in UPF (2-week crossover design)^(39)^ found that an UPF-based diet led to higher energy intake and consequently weight gain compared with the control, i.e., UMPF-based diet. Several prospective cohort studies in adults have also found a connection between higher UPF consumption and increased risk of obesity^(40–42)^. A longitudinal analysis nested in the PREDIMED-Plus trial found that higher consumption of UPF was associated with greater age-related visceral and overall adiposity accumulation^(43)^. Analysis of data from nineteen European countries also showed that the prevalence of obesity at national level was positively correlated with national household availability of UPF^(44)^.

Furthermore, a narrative review on UPF (also defined by NOVA system) applied Bradford-Hill’s criteria to evaluate causality in epidemiological studies linking them to weight gain and obesity^(45)^. Based on these criteria, the evidence in this review showed consistency, temporality, biological gradient, plausibility, coherence and experiment. In contrast to our findings and higher quality studies, cross-sectional studies in other continents have not found strong associations between UPF intake and parameters of obesity and adiposity^(46,47)^. Such variation can be attributed to contextual factors and food system interface, but it should be investigated by future studies.

Of note, the available evidence has shown a link between UPF consumption and increased BMI, but mostly in high-income countries. However, our study adds to the growing body of evidence that UPF consumption is increasingly prevalent among less developed countries of the Mediterranean basin. There is an emerging agreement in the scientific literature (including the results of the present study) that individuals with higher adherence to the Mediterranean diet tend to consume less UPF. For instance, a study in Spain that used the same scoring system (i.e., KIDMED) found that one-third (i.e. 32·2 %) of total energy intake came from UPF and that UPF consumption was inversely associated with higher adherence to the Mediterranean diet^(48)^. This is particularly relevant for Albania, a Mediterranean basin country, where 44 % of its population is estimated to be unable to afford a recommended diet, a higher proportion than any other country in the same modernising and formalising group^(49)^.

Based on our results, eating OH intensity appears to be an important driver of diet, along with the degree of processing. In our analysis, UPF intake was higher among substantial OH eaters and strongly associated with higher BMI, but this association among OH eaters held true also in the case of UMPF. Results from a study in Brazil have suggested the consumption frequency of UPF can be higher when eating OH than when eating AH^(50)^. A study of adolescents in the United Kingdom, based on data from the National Diet and Nutrition Survey Rolling Program, showed that eating AH (*β* = −0·12, 95 % CI (−0·19, −0·05)) was inversely associated with UPF consumption, while fast food consumption (*β* = 0·29, 95 % CI (0·12, 0·47)) was directly associated with increased UPF consumption^(21)^. While cross-sectional studies on the relationship between intake and body size are often hampered by the inaccuracies of food intake measurement methods, larger and better-controlled studies on the topic suggest that eating more UPF away from home is associated with increased BMI and unfavourable changes in body fatness. We speculate that one potential explanation may be that individuals eating more UMPF are eating fewer UPF to compensate for low physical activity or high sedentariness in the potential underlying mechanisms of the association. This warrants further investigation.

It is important to note that the NOVA classification system divides foods into categories based on the extent, nature and purpose of industrial processing. Since its publication in 2010, NOVA has been endorsed by international organisations such as the Pan American Health Organization and UN FAO, as UPF have an impact on not just human health, but also the environment. However, the degree of food processing is not the sole determinant of nutritional quality. In some cases, processing may increase the bioavailability of nutrients and some foods in the NOVA classification can be prepared AH or in industrial settings. While more research is needed in this area, there is adequate evidence to recommend avoidance of UPF in order to optimise health and diet quality, as well as to integrate such narrative in public health nutrition policies and guidelines. Further research on the nutritional values of UPF and UMPF compared with established or emerging nutritional guidelines and standards, as well as their link to risk markers for diet-related non-communicable diseases, is also warranted.

It is timely for policy makers and public health professionals in Albania to address youth nutrition as a priority. This may involve promoting the consumption of UMPF or regulating the availability and affordability of UPF, as well as interventions to reduce their production and supply. Overall, addressing youth nutrition in Albania is crucial for the long-term health and well-being of the country’s young people.

This study provides a first look on UPF intake in a population and setting that has not been studied before, allowing an opportunity to examine the interrelationships between nutritional status, UPF intake and BMI among youth. However, there are some limitations that should be recognised. Our observations are based on a cross-sectional study design and a single multiple-pass 24-h diet recall. While this tool is a valid approach for assessing dietary intake patterns in epidemiological studies, it is important to recognise that this approach may not fully capture long term or seasonal variation in dietary patterns, in the populations under investigation. Previous studies, predominantly among children or adults, found screen time, physical activity, mental health may be correlated with each other, with UPF consumption, and also BMI^(28,51)^. In the critical evaluation of the results, it should be also mentioned that the statistical analysis did not consider potential confounding factors (e.g. physical activity, screen time and the mental health of youth) that may have overestimated the observed associations between UPF and BMI. In addition, recall and classification bias may exist in a self-report survey, but this challenge cannot be fully avoided, and we can assume that the amount of information bias is comparable to that of other similar epidemiological surveys. For instance, depending on the nature of the bias, if participants underreport UPF consumption, but have high BMI, the association would appear weaker and if they overreport, the opposite can happen. Another limitation here is generalisability, as data come primarily from predominantly female university students, which may not be representative of the entire Albanian youth population. However, the data do highlight that dietary quality among youth in university settings is of poor quality, and it is possible that the situation may be worse in the general population. It needs to be stressed that BMI alone is not a reliable indicator of health outcomes, and other factors, such as body fat distribution, also play a significant role. The association of UPF with BMI may be bi-directional, as people with higher BMI may also be more likely to consume UPF, which in turn can further deteriorate their body weight and composition. This possibility should be explored in future, ideally longitudinal studies. An important consideration for such future studies should be incorporating UPF, weight history, body composition, clinical outcomes and undermeasured consequences of weight gain (e.g. psychological toll of obesity), to fully characterise diet–BMI–health interactions.

## Conclusion

Our findings provide evidence for the hypothesis that eating OH plays an important role in the association of UPF and UMPF with BMI, while showing that UPF are unfavourably linked to BMI. While causality could not be established due to cross-sectional design, to the best of our knowledge, we provide a first assessment of UPF and UMPF intake in south-eastern Europe, while highlighting the need for establishing and integrating youth nutrition into national surveillance and monitoring systems for key dietary risk factors in Albania. Our design can be used as baseline for future studies investigating malnutrition in all its forms and the role of UPF in this region. Although more research is needed to better understand the role of UPF in body weight and composition changes, we advocate for the inclusion of diet’s degree of processing in nutrition discourse and guidance targeting youth nutrition, particularly in the context of Albania.
